# Temperature and Lateral Pressure Sensing Using a Sagnac Sensor Based on Cascaded Tilted Grating and Polarization-Maintaining Fibers

**DOI:** 10.3390/s24216779

**Published:** 2024-10-22

**Authors:** Yifan Liu, Yujian Li, Pin Xu, Changyuan Yu

**Affiliations:** 1 Department of Electrical and Electronic Engineering, The Hong Kong Polytechnic University, Hong Kong, China; funtata.liu@connect.polyu.hk (Y.L.); 21041651r@connect.polyu.hk (Y.L.); 23132287r@connect.polyu.hk (P.X.); 2 Photonics Research Center, The Hong Kong Polytechnic University Shenzhen Research Institute, Shenzhen 518000, China

**Keywords:** Sagnac loop, TFBG, PMF, temperature, lateral pressure

## Abstract

This study introduces a Sagnac Interferometer (SI) fiber sensor that integrates Polarization-Maintaining Fibers (PMFs) with a Tilted Fiber Bragg Grating (TFBG) for the dual-parameter measurement of strain and lateral pressure. By incorporating a 6° TFBG with PMFs into the SI sensor, its sensitivity is significantly enhanced, enabling advanced multi-parameter sensing capabilities. The sensor demonstrates a temperature sensitivity of −1.413 nm/°C and a lateral pressure sensitivity of −4.264 dB/kPa, as validated by repeated experiments. The results exhibit excellent repeatability and high precision, underscoring the sensor’s potential for robust and accurate multi-parameter sensing applications.

## 1. Introduction

Lateral pressure sensors are integral to evaluating structural integrity across various engineering applications, particularly in fields such as structural health monitoring [1], geotechnical engineering [2], and aerospace systems [3]. These sensors play a critical role in accurately measuring the lateral stresses exerted on structural elements, ensuring their operational safety and longevity. The design requirements for lateral pressure sensors include heightened sensitivity, rapid response time, and robustness in harsh environments. Various design schemes have been proposed, with optical fiber sensors emerging as the preferred solution due to their immunity to electromagnetic interference, compact form, and ease of integration.

Due to the relatively complex environment of lateral pressure monitoring, simultaneous temperature measurements during lateral pressure testing are highly meaningful. Optical fiber-based lateral pressure sensors encompass a range of design approaches, each presenting distinct advantages and limitations. Fiber Bragg gratings (FBGs) [4,5] offer exceptional sensitivity and precision but may have a limited dynamic range and susceptibility to cross-sensitivity. Fabry–Perot Interferometer (FPI) [6], Mach–Zenhder Interferometer (MZI) [7], and Michelson interferometer (MI) sensors [8] provide heightened sensitivity and versatility but may require complex fabrication processes and be susceptible to temperature-induced drift. Microbend sensors [9] offer simplicity and cost-effectiveness but may suffer from limited sensitivity. A pair of fiber Bragg gratings embedded in a polyurethane diaphragm [10] was fabricated to reduce the crosstalk effect of temperature. However, this process resulted in a complex demodulation procedure and could not support a simultaneous temperature measurement. Optical fiber sensors based on attachment materials like PDMS [11] and PVA [12] can offer high temperature sensitivity. However, the difficulty in their fabrication and their low reproducibility limit their application. In this study, a sensor capable of simultaneously monitoring temperature and lateral pressure with high sensitivity using a combination of tilted gratings and a Sagnac Interferometer is achieved.

The Sagnac Interferometer (SI)’s structure uses a prominent interferometric sensor design in optical fiber sensing and comprises a coupler and a high-birefringence fiber. This structure has undergone extensive development by researchers over many years for sensing temperature [13], strain [14], and curvature [15]. SI sensors based on polarized-mode coupling have undergone significant development for stress and pressure measurements. High-birefringence loop mirrors have been interpreted and applied in various sensor applications [16]. Building upon this, a combination of polarization-maintaining fibers (PMFs) and commercial FBGs was incorporated into the Sagnac loop for highly sensitive strain measurements [17].

Tilted Fiber Bragg Grating (TFBG), akin to FBG, features a structure in its optic fiber core consisting of a series of gratings with uniform refractive index changes over the same period. Unlike in FBG, in TFBG, the radial direction of the fiber is inclined at a specific angle [18]. When light propagates in the cylindrical waveguide of the fiber core, it couples backward into the cladding due to the tilted grating region in the core, thus forming a series of resonance modes. Among these, the core-mode properties in the fiber core are similar to those of FBGs, such as excellent sensing properties for temperature [19] and strain [20]. Due to variations in the grating region, the phase-matching conditions change, leading to a drift in the resonance wavelength, enabling stable and highly sensitive sensing. Additionally, when the grating region experiences changes in lateral pressure, the stress distribution in the fiber core changes [21,22], affecting the coupling coefficient between the core light and the cladding light, as well as the mode field size. This results in changes in the transmission intensity of the resonance modes, which allow the sensor to detect lateral pressure. Moreover, since the intensity changes of these resonance modes are not sensitive to temperature [23], this provides the sensor with the ability to perform multi-parameter measurements.

## 2. Fabrication and Methodology

### 2.1. Sensor Fabrication

The SI [13] operates based on the principle of interference, where a beam of light is split into two paths that travel in opposite directions around a loop. When the loop rotates, a phase shift occurs between the two beams due to the difference in their travel times, which can be detected to measure rotational motion or other parameters. This sensor is based on a 1:1 coupler and high-birefringence optical fiber to realize a Sagnac ring for a highly sensitive vibration sensor. When light from a broadband source passes through the coupler, it is split 1:1 and then travels through both the clockwise and counterclockwise paths before being collected by the coupler again. The collected beams interfere with each other, and the transmitted spectrum is obtained through the output fiber as an interference pattern. In this study, commercial panda polarization-maintaining fibers are used for the sensor design. Additionally, a 6° tilted grating is cascade-spliced with a polarization-maintaining fiber core and connected to the Sagnac sensor.

A 6° tilted grating is an appropriate choice for the tilt angle because such a tilted grating exhibits a high core-mode resonance intensity and a clear cladding mode region, making it suitable for multi-parameter sensing. A 213 nm solid-state laser was utilized for the fabrication process. The laser passed through a 535.9 nm phase mask and was directed onto a hydrogen-doped fiber. The first-order interference fringes generated by the phase mask acting on the optical fiber enable the writing of a grating region with a regularly changing refractive index. It is crucial to maintain proper alignment between the phase mask and the optical fiber throughout this process. In this study, a 12 mm region was inscribed on an single-mode fiber using a 53 mW laser output and a writing speed of 0.01 mm/s. It has been verified that, based on the fabrication parameters, the core-mode resonance amplitude of this tilted grating sensor exceeds 20 dB while also possessing excellent cladding resonance mode characteristics typical of a 6° tilted grating, with clear ghost modes and cladding mode regions.

**Figure 1:**
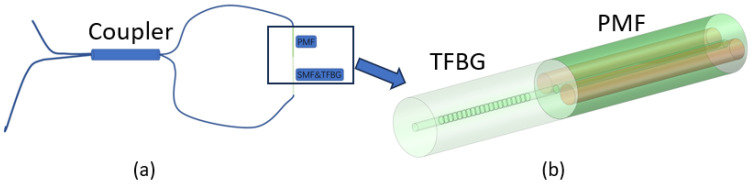
(**a**) Structure of the core-to-core linking of TFBG and PMF. (**b**) Structure of the designed sensor.

PMF and single-mode fibers containing tilted gratings are core-to-core connected. A 1:1 2 × 2 coupler was used to fabricate the sensor. A 20 cm section of polarization-maintaining fiber was used in the fabrication of the sensor, based on the light source bandwidth and the interferometer’s FSR. One end of the coupler was used as an input connected to a broadband light source, while the other end was connected to an optical spectrum analyzer (OSA, Yokogawa AQ6370D) (“Yokogawa”, Aira District, Tokyo, Kagoshima Prefecture, Japan) for the output. The two output ports of the coupler were core-to-core connected to the ends of the PMF and tilted grating fibers, thus forming a SI sensor. The high-birefringence characteristics of the PMF and the birefringence properties of the tilted grating under compression provide higher temperature sensitivity. See Figure 1.

### 2.2. Methodology

The wavelength of the coupled mode of TFBG λ can be calculated by

(1) $$\lambda^{core}=2n_{core}\Lambda .$$

(2) $$\lambda^{i}=\left(n_{core}+n_{clad}^{i}\right)\Lambda /cos\theta .$$

where Λ is the grating period. In this study, a 535.9 nm phase mask was used to write the grating. *n* is the order of the mode in the transmission spectrum. With the phase-match condition theory, the interference transmission spectrum can be interpreted as follows [18]:

(3) $$T=\frac{1-cos(\varphi )}{2}\;,$$

where φ can be calculated as

(4) $$\varphi =\frac{2\pi (B\left(\lambda \right)L_{P}-\frac{2NL_{T}}{\cos(\delta )})}{\lambda }\;,$$

where *B* is the birefringence of a PMF fiber with a length of $L_{T}$. δ is the angle of the grating. By combining this with the phase-match condition,

(5) $$\frac{2\pi (B\left(\lambda \right)L_{P}\cos\left(\delta \right)-2N_{eff}L_{T})}{\lambda \cos(\delta )}=2m\pi \;m=0,\pm 1,\pm 2,\ldots ,$$

Subsequently, by taking the derivative of sensitivity *S* with respect to temperature *t*, we obtain the following formula. Therefore, the sensitivity parameter S can be written as follows [13]:

(6) $$S=\frac{d\lambda }{dt}=\frac{\lambda (\frac{\partial B}{\partial t}L_{P}+B\frac{dL_{P}}{dt}\cos\left(\delta \right))}{\left(B\left(\lambda ,t\right)L_{P}\left(t\right)-\lambda \frac{\partial B}{\partial t}L_{P}\right)\cos\left(\delta \right)-2N_{eff}L_{T}}\;$$

When the parameter *S* is compared with the sensitivity without TFBG $S_{1}$, it can be written as

(7) $$S_{1}=\frac{d\lambda }{dt}=\frac{\lambda (\frac{\partial B}{\partial t}L_{P}+B\frac{dL_{P}}{dt})}{B\left(\lambda ,t\right)L_{P}\left(t\right)-\lambda \frac{\partial B}{\partial t}L_{P}}\;$$

From the above derivation, it can be seen that when the TFBG is introduced into the SI, the sensitivity of the sensor changes. The sensitivities before and after adding the TFBG are represented by $S_{1}$ and *S*, respectively. Comparing the two sensitivities, it is clear that $S_{1}<S$. Therefore, an enhancement in the sensitivity can be found by adding the TFBG.

The tilted grating structure breaks the cylindrical symmetry of the optical fiber, inducing a birefringence that is absent in conventional fiber gratings. Consequently, tilted grating was selected for this study to leverage these unique properties. When lateral pressure acts on the tilted grating region, the grating region will generate symmetric compressive stress in different directions, resulting in changes in the fiber’s photoelastic coefficient. According to the coupling coefficient [24,25],

(8) $$\kappa_{i,core}=C\int \int_{-\infty }^{+\infty }\left(E_{x}^{i}E_{x}^{core*}+E_{y}^{i}E_{y}^{core*}\right)\Delta ndxdy.$$

When fluctuations occur in optical coupling coefficients under applied pressure, they lead to significant changes in the resonance peak intensity. As the optical fiber undergoes deformation due to compression, the intensity of the transmission spectrum’s resonance peak can be utilized for lateral pressure sensing. Due to the complexity of the optical coupling intensity model, demonstrating the usability of this sensor requires repeated experiments and trials with various samples. In this study, multiple sets of samples were prepared to validate the repeatability and consistency of the sensor.

## 3. Experiment and Discussion

### 3.1. Temperature Measurement

**Figure 2:**
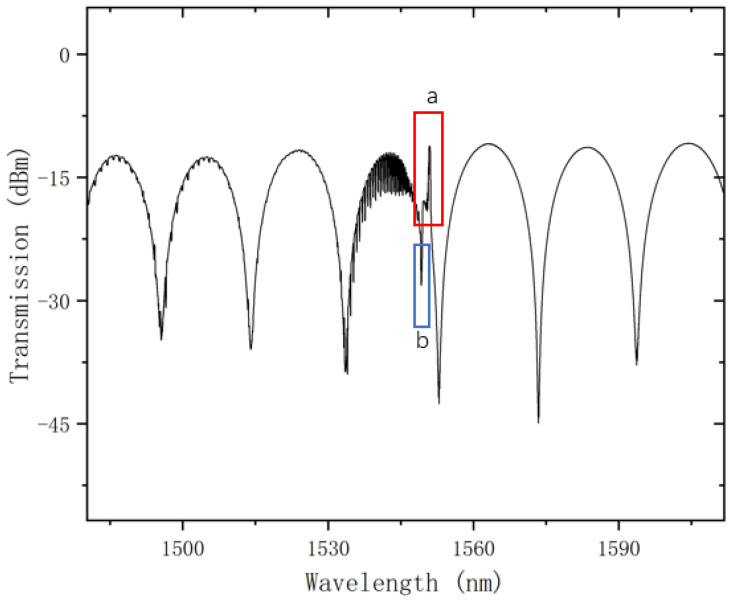
Transmission spectrum of SI combined with TFBG.

The spectrum of the fabricated sensor is shown in Figure 2.

From the transmission spectrum, one can observe an upward peak labeled “a” and a downward dip labeled “b” around 1550 nm. From a wavelength perspective, they would be closer to the core-mode and cladding-mode regions. These regions are sensitive to lateral pressure on the optical fiber. Additionally, one can observe a series of resonance peaks coupled onto the large envelope formed by the Sagnac ring. Clearly, these resonance peaks originate from the cladding modes of the tilted grating.

**Figure 3:**
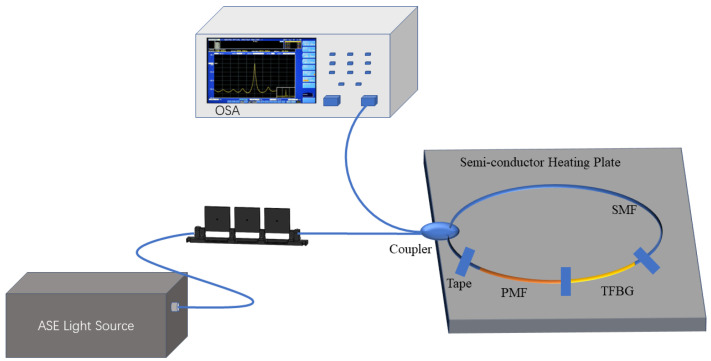
Temperature measurement experiment.

This research experiment utilized a C + L band Amplified Spontaneous Emission (ASE) light source emitting light in the range of 1480 nm to 1620 nm. The experimental setup is illustrated in Figure 3.

**Figure 4:**
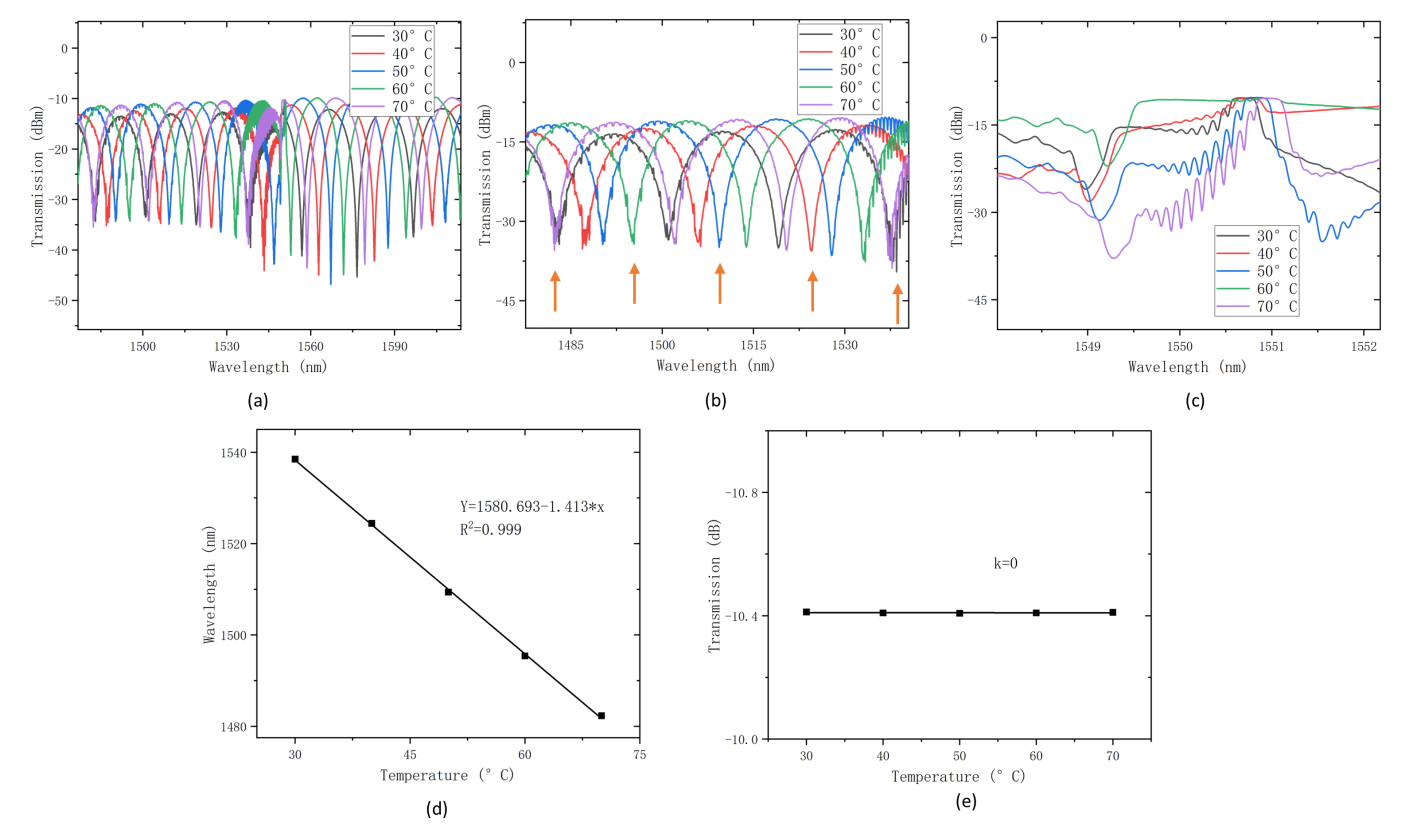
(**a**) Obtained transmission spectrum, (**b**) spectrum at 47 °C, (**c**) detailed graph of peak 1, and (**d**) linear fit of temperature sensing. (**e**) Intensity change of Peak A with temperature variation).

The transmitted spectrum was captured using an OSA with a minimum resolution of 0.02 nm. To ensure the secure attachment of the birefringence fiber and TFBG to the heating plate, both edges of the PMF-TFBG combination were taped onto the surface of the plate. Given the heating range of the semi-conductor heating plate is stable from 30 °C to 70 °C, a sensing temperature range of 30 °C to 70 °C was selected. Based on the polarization characteristics of the tilted grating, the incident light in this study was controlled to be S-polarized. As a solid metal heating plate may not always provide stable and linear heating, additional time was needed for temperature stabilization during the heating process to ensure data accuracy. By heating the heating plate in 5 °C increments, the sensor’s temperature measurement values could be obtained.The results are depicted in Figure 4.

Figure 4a depicts the variation in the transmission spectrum of the sensor with temperature changes. We extracted the spectrum within the range of 1480–1550 nm and selected the interference dip at 1538.49 nm at 30 °C as the sensing feature for investigation, as illustrated in Figure 4b. It is observable that, with increasing temperature, the wavelength of the dip in this envelope undergoes a blue shift, indicating a shorter wavelength. Through the extraction of wavelength data for this dip and a subsequent regression analysis, as depicted in Figure 4d, we obtained a function describing the dip wavelength’s dependency on temperature: Y = 1580.693 − 1.413 × x. This implies a sensitivity of 1.413 nm/°C for temperature, with a high regression confidence coefficient of R = 0.999.

Conversely, we examined the peak at 1550.66 nm to assess its response to temperature changes. We found that the wavelength variation of this peak exhibited a low sensitivity to temperature, manifesting as a subtle red shift. Simultaneously, we emphasized the investigation of the peak’s transmission intensity, as illustrated in Figure 4e. It is evident that the transmission intensity of this peak remains relatively constant across temperature changes. This property is advantageous, indicating that when lateral pressure is applied to the sensor, selecting this peak as the sensing feature is appropriate as it is not susceptible to temperature interference, thereby achieving interference-free multi-parameter measurements.

### 3.2. Lateral Pressure Measurement

**Figure 5:**
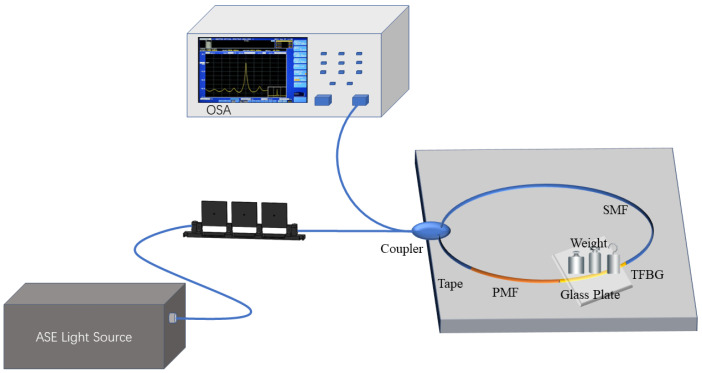
Lateral pressure measurement experiment.

As depicted in Figure 5, the experimental system still employed a C + L band ASE light source emitting light in the range of 1480 nm to 1620 nm. The flat plate area for securing the fiber was 12 cm × 12 cm, which is capable of exerting pressure on the lateral direction of the fiber. A glass plate was pressed onto the flat plate, sandwiching the sensor between them. With the pressure from the glass plate and the counterweight, taping the sensor to the flat platform was no longer necessary. In this study, ten 100 g balance weights were utilized, with a chosen sensing range of 1000 g and a sensing resolution of 100 g.

**Figure 6:**
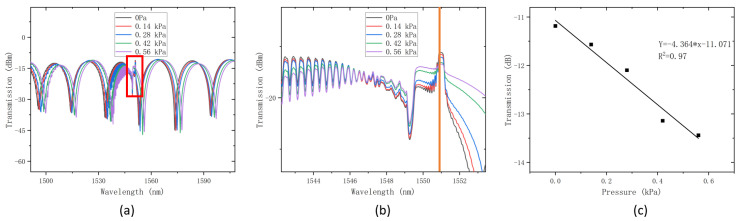
(**a**) Obtained transmission spectrum, (**b**) detailed graph of peak at 47 °C, and (**c**) linear fit of temperature sensing.

According to the pressure formula

(9) $$P=\frac{F}{S}\;,$$

where *F* is the stress by weight and S is the pressure area, and based on a square area with a lateral pressure application surface of 12 cm × 12 cm, the area of this region is 0.014 m^2^. When the mass of the weights is 0, 200 g, 400 g, 600 g, and 800 g, the corresponding pressures are 0, 0.14 kPa, 0.28 kPa, 0.42 kPa, and 0.56 kPa, respectively. The results of the lateral pressure measurements are illustrated in Figure 6.

From the graph of the results, it can be observed that the variation of the peak in the transmission spectrum at the wavelength of 1550.9 nm reveals the following: when lateral pressure is applied to the grating region, there is no change in the position of the peak wavelength. However, as the lateral pressure increases, the transmission intensity of the peak decreases. After extracting and linearly fitting the transmission intensity of this peak, as depicted in Figure 6c, we obtained a function representing the relationship between the lateral pressure and the transmission intensity of this peak at 1550.9 nm: Y = −4.264 × x − 11.071. The confidence factor is R = 0.97. Consequently, the sensitivity of lateral pressure sensing based on transmission intensity at this wavelength for this sensor is −4.264 dB/kPa.

### 3.3. The Simultaneous Measurement of Lateral Pressure and Temperature

As shown the results and discussion above, the demonstrated sensor device enables the simultaneous measurement of lateral pressure and temperature. It allows for the establishing of a matrix-based relationship between lateral pressure and temperature, which expresses the interdependence of these two parameters as follows [10]:

(10) $$\left(\begin{array}{c} \Delta P\\ \Delta T \end{array}\right)=\varepsilon \left(\begin{array}{cc} K_{PP} & K_{PT}\\ K_{TP} & K_{TT} \end{array}\right)\left(\begin{array}{c} \Delta p\\ \Delta t \end{array}\right)$$

where ΔP and ΔT represent the variation in lateral pressure and temperature. Δp represents the variation of the intensity with a lateral pressure change. Δt denotes the wavelength shift of the chosen interference spectrum dip. The matrix coefficients $K_{PP}$, $K_{PT}$, $K_{TP}$, and $K_{TT}$ correspond to the crossover factors, respectively. Based on our experimental findings, $K_{PT}=k_{TP}=0$, the matrix in Equation (10) can be derived as

(11) $$\left(\begin{array}{c} \Delta P\\ \Delta T \end{array}\right)=\varepsilon \left(\begin{array}{cc} -1.413 & 0\\ 0 & -4.264 \end{array}\right)\left(\begin{array}{c} \Delta p\\ \Delta t \end{array}\right)$$

By utilizing this matrix equation, the resolution for temperature was −1.413 nm/°C. We also achieved a lateral pressure sensitivity of −4.264 dB/kPa.

### 3.4. Repeated Experiments

Given that the sensor utilizes measurements of transmission spectrum intensity, and considering the complexity of the transmission intensity model, repeated experiments are crucial. They serve as the cornerstone for the reproducibility of the sensor’s results. In this study, the experiments on lateral pressure were repeated three times. The sensitivities obtained were −4.261 dB/kPa, −4.267 dB/kPa, and −4.262 dB/kPa, respectively. The sensor exhibited excellent repeatability.

## 4. Conclusions

This study developed a sensor capable of simultaneously measuring temperature and lateral pressure with high sensitivity. The sensor combines a 6° tilted grating with a segment of commercially available panda polarization-maintaining fiber, connected in series and linked to two ports of a 2 × 2 coupler at one end. This setup enables the concurrent measurement of lateral pressure and temperature. By incorporating a broadband light source, the sensor generates an interference spectrum coupled with a tilted grating spectrum. When the temperature of the environment changes, the interference envelope of the sensor’s transmission spectrum undergoes a blue shift towards shorter wavelengths at a sensitivity of −1.413 nm/°C. During this process, the transmission peak at 1550.6 nm undergoes a slight red shift with no change in transmission intensity. When lateral pressure is applied to the grating region, the intensity of this transmission peak varies at a sensitivity of −4.264 dB/°C. Due to the absence of crosstalk between these two features, simultaneous measurements can be achieved.

## Data Availability

The original contributions presented in the study are included in the article, further inquiries can be directed to the corresponding author.
